# Dynamics of indicators of mental health and mental wellbeing among university students during the first year of the COVID-19 pandemic

**DOI:** 10.1192/j.eurpsy.2022.1280

**Published:** 2022-09-01

**Authors:** L. Shaigerova, O. Almazova, A. Dolgikh, Y. Zinchenko

**Affiliations:** Lomonosov Moscow State University, Psychology, Moscow, Russian Federation

**Keywords:** university students, Covid-19, mental health, mental well-being

## Abstract

**Introduction:**

University students’ lifestyle has changed dramatically due to the spread of COVID-19. They had to face adaptation to the online learning format, as well as strict and long-term restriction of social contacts.

**Objectives:**

To trace the dynamics in the main indicators of mental health (depression, anxiety, and stress) and mental wellbeing among students during the first year of the COVID-19 pandemic.

**Methods:**

DASS (Lovibond, Lovibond, 1995) and WEMWBS (Tennant et al., 2007) were applied in the research. The study involved 733 students at Russian universities aged from 18 to 23 years (M=20.0; SD=3.23), of which 88.1% were girls. The same design was used in the spring (N=245), in the autumn of 2020 (N=270) and in the winter of 2021 (N=218).

**Results:**

It was found that the indicators for all DASS scales significantly differ (p<0.05) across the three periods. With Post Hoc Scheffe, it was shown that the levels of depression, anxiety and stress in autumn 2020 and winter 2021 were significantly higher than in spring 2020 (p<0.05). The WEMWBS values differ significantly (p<0.05) throughout the periods. The level of mental wellbeing among students in spring 2020 was significantly higher than in autumn 2020 and in winter 2021 (p<0.05).

**Conclusions:**

It was revealed that despite the rather severe restrictions in Russia at the beginning of the pandemic, and relatively mild measures taken afterwards (compared to many countries), the indicators of students’ mental health as well as the level of their mental well-being continued to decline. The reported study was funded by RFBR, project number 20-04-60174.

**Disclosure:**

No significant relationships.

